# “Your Skin Crawled Every Time He Touched You”: A Secondary Qualitative Analysis Exploring Bagwell-Gray's Taxonomy of Intimate Partner Sexual Violence

**DOI:** 10.1177/10778012231174352

**Published:** 2023-05-19

**Authors:** Leslie M. Tutty, Cindy Ogden, Kendra L. Nixon

**Affiliations:** 1 Faculty of Social Work, University of Calgary, Calgary, AB, Canada; 2 Private Practice, Calgary, AB, Canada; 3 Faculty of Social Work, University of Manitoba, Winnipeg, MB, Canada

**Keywords:** intimate partner sexual violence, sexual assaults by partners, sexual coercion, secondary qualitative analysis

## Abstract

Bagwell-Gray et al. developed a taxonomy of intimate partner sexual violence (IPSV) focusing on type of force (physical or nonphysical) and type of sexual activity (penetration or no penetration). The current secondary descriptive qualitative analysis of interviews with 89 Canadian women IPV victims assessed IPSV that fit Bagwell-Gray's taxonomy. About half (46 or 51.7%) described sexual violence, most commonly sexual abuse (26 or 29.2%), sexual assaults (17 or 19%), and sexual coercion (16 or 17.9%), with overlap across categories. Forced sexual activity was seldom mentioned (3 or 3.4%). Implications for service providers and researchers are provided.

The seriousness and widespread nature of intimate partner violence (IPV) has been acknowledged globally (García-Moreno & Pallitto, 2013). The nature of IPV is often considered to be primarily physical, and potentially life-threatening. However, while the risk of IPV homicide remains of significant concern, a relatively small number of women are murdered by partners (Dawson et al., 2009). Other common forms of IPV include psychological abuse or coercive control (Ansara & Hindin, 2011; Sharp-Jeffs et al., 2018), financial abuse (Krigel & Benjamin, 2021), and sexual assaults (Tarzia, 2021a; Tarzia & Tyler, 2021; Tutty & Nixon, 2022), the focus of the current study.

The extent to which IPV includes sexual assaults and other sexual victimization needs further elaboration (Bagwell-Gray et al., 2015; Logan et al., 2007; Wright et al., 2021). Intimate partner sexual violence (IPSV), the broadest term, occurs frequently. In the 2015, U.S. National Intimate Partner and Sexual Violence Survey (Smith et al., 2018), with respect to just one aspect of IPSV, contact sexual violence by partners (IPSA), almost one-fifth (18.3%) of American women reported this.

The bulk of IPSV research has focused on IPSA, originally titled “marital rape” (Bergen, 2004; Russell, 1982). Male partners who sexually assault women also commonly abuse them in multiple ways such as physical abuse and stalking (Basile, 2008; Krebs et al., 2011). In general, the nature of the physical partner abuse in women whose partners sexually assault them is more severe (Black et al., 2001; Symes et al., 2014). IPSA also commonly occurs after separation (DeKeseredy & Joseph, 2006; Rennison et al., 2012).

The consequences of partner sexual assaults have been documented, often in comparison to other forms of IPV, including more pronounced effects on physical health (Bonomi et al., 2007) and PTSD (Bennice et al., 2003; McFarlane et al., 2005, Temple et al., 2007). In research examining mental health issues in addition to PTSD, women whose partners sexually assaulted them also reported significant depression, anxiety, and other difficulties such as suicidality (Honda et al., 2018; Tarzia et al., 2018). Tutty and Nixon (2022) reported that abused women who had experienced any partner sexual assaults had endured significantly more severe partner violence, mental distress, and depression than other IPV survivors. PTSD was more severe for women with both IPSA and child sexual abuse histories.

The concept of sexual violence has been broadened to include aspects beyond sexual assaults. The most commonly studied is sexual coercion, which is a subset of coercive control (Mitchell & Raghavan, 2021). Stark (2007) defined coercive control as “the use of force or threats to compel or dispel a particular response” (p. 228). Sexual coercion includes tactics such as pressure, emotional force, or trickery and threats to have sex such as, “I’ll leave if you don’t sleep with me.” The mental health consequences of sexual coercion have not been established as, for example, several studies failed to link sexual coercion and PTSD (Basile et al., 2004; Mechanic et al., 2008). Reproduction coercion or controlling a partner's use of birth control or pregnancy has also received research interest (Bagwell-Gray et al., 2021; Grace, 2018; Thaller & Messing, 2016).

In a review of factors associated with IPSV, Logan et al. (2015) added sexual degradation and humiliation as important behaviors to assess in addition to sexual assaults and sexual coercion. These authors also highlighted a significant gap in knowledge about the full scope and nature of IPSV. Bagwell-Gray et al. (2015) provided the first comprehensive model of the variety of ways that intimate partners sexually violate women, the focus of the next section.

## Bagwell-Gray's Taxonomy of IPSV

Bagwell-Gray et al. (2015) developed a unique taxonomy of forms of IPSV that vary in terms of the type of force used (physical or nonphysical) and the type of sexual activity (penetration or no penetration), defined as follows:

The level of forcefulness refers to the degree of physical force used, ranging from physical violence at the high end of physical force to nonphysical manipulation at the low end of physical force. The level of invasiveness refers to how invasive the type of sexually abusive act is, ranging from vaginal, oral, or anal penetration at the high end of invasive acts to unwanted touching at the low end of noninvasive acts. (p. 324)

The resulting four forms are sexual assault (high force and high invasiveness), sexual coercion (low force and high invasiveness), forced sexual activity (high force and low invasiveness), and sexual abuse (low force and low invasiveness). To validate the taxonomy, Bagwell-Gray (2021) interviewed 28 women IPV survivors who had not necessarily disclosed sexual assaults by their partners. They were asked explicitly about the sexual behaviors in their relationships, which were analyzed to determine whether they fit the taxonomy. These results are presented in the next sections that detail the forms of sexual violence.

Intimate partner sexual assault (high force and high invasiveness) refers to:

the use of physical violence or the threat of physical violence to obtain, or attempt to obtain, unwanted oral, vaginal, or anal intercourse, including forced penetration and sexual assault with objects. This term also applies to unwanted penetration when a victim/survivor is unable to consent or is unaware, that is, asleep or under the influence of drugs and alcohol. (Bagwell-Gray et al., 2015, p. 324)

In Bagwell-Gray's study (2021), 14 or half of the 28 women reported partner sexual assaults. While sexual assaults may seem the most obvious IPSV form to identify, about 80% of the 208 women in a study by Jaffe et al. (2021) did not perceive sexual assaults by an intimate partner as “rape.” Logan et al. (2015) suggest several reasons for this, including that rape is supposedly enacted by strangers and that sex is a “spousal duty” (see also Tarzia, 2021b; Wright et al., 2021).

While similar to intimate partner sexual assault in that penetration occurs, intimate partner sexual coercion (low force and high invasiveness) is not physically forced. Rather, “the unwanted sex act is obtained through manipulative tactics and control” (Bagwell-Gray et al., 2015, p. 324), such as intimidation, verbal manipulations, threats to humiliate, or withholding essential resources. It also includes the partner forcing her to have sex with an additional person, although not many other authors mention this, Logan et al. (2007) being an exception. In Bagwell-Gray's (2021) study, 19 women (68%) reported sexual coercion. Given the previously mentioned phenomenon of women not identifying sexual assaults by their partners as rape, disregarding the importance of sexual coercion should not be surprising. Other research specific to this includes Goetz and Shackelford (2009), Jeffrey and Barata (2017), Snead and Babcock (2019), and Starratt et al. (2008), each of which highlighted the serious nature of sexual coercion.

Intimate partner-forced sexual activity (high force and low invasiveness) is defined as “nonpenetrative sexually abusive acts that are physically forced” (Bagwell-Gray et al., 2015, p. 325) such as being held down and masturbated upon or physical violence toward a sexual body part. Bagwell-Gray et al. note that this has rarely been documented in the literature, clarifying that the sexual nature of the behaviors may result in few disclosures. Originally developed theoretically from the circumplex of force versus nonforce and high intrusiveness versus low intrusiveness, in Bagwell-Gray (2021) study, only the experiences of two women (7%) fit the forced sexual activity category.

Intimate partner sexual abuse (low force and low invasiveness) includes male partners refusing to use condoms and birth control sabotage (reproductive coercion), male partners having sex outside the primary relationship, and exerting control over sexual decision-making. Other examples include sexual insults (e.g., “you’re a whore”) and forcing a partner to watch pornographic material. Reproductive coercion has received attention (Bagwell-Gray et al., 2021); Grace, 2018; Thaller & Messing, 2016). Reproductive coercion is defined as “birth control sabotage, pregnancy coercion, or controlling the outcome of a pregnancy” (Grace, 2018, p. 371). Sex outside the relationship, or a partner's infidelity, is a risk for women, not only with respect to the betrayal of the partner relationship, but also because of the possibility of the man developing sexually transmitted diseases and infecting their partners (Hess et al., 2012; Utley, 2017).

Bagwell-Gray et al. (2015) noted that none of the examples in the sexual abuse category would be considered criminal acts under American law. In Bagwell-Gray's (2021) study, 27 women or 96% of the IPV respondents reported incidents congruent with sexual abuse.

In summary, despite the seeming utility of Bagwell-Gray's taxonomy, little research specific to this has been published although we note that the model is only 7 years old. As mentioned, to assess the taxonomy fit, Bagwell-Gray (2021) interviewed 28 women who were IPV victims. Fernet et al. (2021) interviewed 71 young Canadian women in dating relationships (not specified as violent), 29.6% of whom reported sexual IPV in the past year, with Bagwell-Gray's taxonomy reflected in their experiences. Although dating relationships can be arguably different from live-in committed long-term relationships (Sutton & Dawson, 2021), these young women identified that intimate partner sexual coercion (20 or 28.2%), sexual assaults (10 or 14.1%), sexual abuse (9 or 12.7%), and forced sexual activities (5 or 7%) had all occurred.

Further research with respect to various forms of IPSA is clearly warranted. With unique access to interviews with women who participated in a large study of Canadian women who were abused by intimate partners, the goal of the current secondary qualitative data analysis was to examine the fit of Bagwell-Gray's taxonomy.

## Method

This article reports on a secondary descriptive qualitative data analysis undertaken with 89 women interview participants from a longitudinal study entitled the “The Healing Journey” (Tutty, Radtke, Ateah et al., 2021; Tutty, Radtke, Thurston et al., 2021). The original study recruited a convenience sample of 665 Canadian women abused by intimate partners who had sought formal support in the Canadian provinces of Alberta, Saskatchewan, and Manitoba. Longitudinal data were collected in seven waves between 2005 and 2009. The criteria for inclusion were: a minimum of 18 years of age; the most recent incident of IPV no sooner than three months and no longer than five years prior; commitment to stay in the study for the full four years; and no significant mental health issues.

In addition to the longitudinal quantitative focus, the researchers developed a qualitative component inviting the women to tell their stories from their own perspectives. The semi-structured interviews were designed by a subgroup of Healing Journey team members, with the intention of gaining depth and context to the respondents’ experiences of IPV. The research protocols for both the original and the qualitative interview component were approved by the Ethical Review Boards of the six associated universities (Universities of Calgary, Manitoba, Regina, Brandon, Lethbridge, Winnipeg). Each woman was asked to begin where they thought their journey/story of IPV began, where that journey/story is at today, and where they think it is taking them in the future. Notably, no interview question was specific to any form of sexual assault within the relationships. Honoraria of $50 CAN was provided to participants at each wave and for the one-time qualitative interviews.

The qualitative interviews were conducted in 2007 and 2008, about halfway through the longitudinal study. The interviews took approximately 2–2.5 hr, scheduled at times and locations convenient to the participants. The research assistants who had administered the quantitative component at each wave were trained to conduct qualitative interviews. The interviews were audiotaped and transcribed verbatim.

### Demographics of the Research Participants

In total, 89 interviews were completed (30 women from Manitoba, 30 from Saskatchewan, and 29 from Alberta). As is evident from Table 1, more than half of the 89 women interviewed were of Indigenous origin (49 or 55.1%; First Nations 38 or 32.7%; Métis 10 or 11.2%; Inuit 1 or 1.1%), with a little more than a third White (*n* = 31 or 34.8%), and a much smaller proportion of visible minority status (9 or 10.1%; Latin American 5 or 5.6%; South Asian 2 or 2.2%; Black = 1 or 1.1%; Filipino = 1 or 1.1%). The racial backgrounds of the partners were 50% White, 42.3% Indigenous, and 6.8% visible minority.

**Table 1.** Women's Demographic Profile (*N* = 89).

Variable	Categories	Means/frequency
Age (*N* = 89)		39.8 (*SD* = 11.6)
Racial/ethnic background (*N* = 89)	White	31 (34.8%)
	Indigenous	49 (55.1%)
	Visible minority	9 (10.1%)
Sexual orientation (*N* = 89)	Heterosexual	79 (88.8%)
	Bisexual	5 (5.6%)
	Lesbian	3 (3.4%)
	Two-spirit	2 (2.2%)
Partner's age (*N* = 88)		41.3 (*SD* = 12)
Current partner relationship (*N* = 89)	No longer together	64 (71.9%)
	Together	25 (28.1%)
Partner's racial/ethnic background (*N* = 88)	White	44 (50%)
	Indigenous	48 (43.2%
	Visible minority	6 (6.8%)
Length of relationship in years (*N* = 89)		10.6 years (*SD* = 10.2)
Length relationship was abusive (*N* = 86)		8.8 years (SD = 9.6)
Children? (*N* = 89)	Yes	79 (88.8%)
	No	10 (11.2%)
Total income in past year (*N* = 79)		$29,436 (*SD* = $31,335)
Highest education (*N* = 89)	Not completed HS	26 (29.2%)
	Completed HS or GED	19 (21.3%)
	Post-secondary-technical	15 (16.9%)
	Post-secondary-university	29 (32.6%)
Currently employed (*N* = 88)	Full-time	28 (31.5%)
	Part-time/casual	19 (21.3%)
	Not working	41 (46.1%)
Any child sexual abuse history (*N* = 89)	No child sexual	43 (48.3%)
	Child sexual abuse	46 (51.7%)
Ever had sex against will? (*N* = 89)	Yes	68 (76.4%)
	No	21 (23.6%)

Most women were heterosexual (*n* = 79), with five women identifying as bi-sexual, three as lesbian, and two as Two-Spirit. The majority (71.9%) no longer lived with the partner who abused them. In terms of education, about half (49.5%) had some form of post-secondary education, while 30% had not completed high school. A little less than one-third were working full-time (31.5%), 19% worked part-time/casual (21.3%), and 46.1% were not currently employed. More than half (51.7%) reported a child sexual abuse history and 76.4% reported having had “sex against their will,” a category that refers to child sexual abuse, IPSA, and sexual assaults from other, unspecified assailants.

### Quantitative Research Measure

The nature of the IPSV was assessed using the Composite Abuse Scale (CAS; Hegarty et al., 2005). This IPV screening measure consists of 30 items, rated for frequency in the past 12 months on a 6-point scale from *never* to *daily*, with a possible total of 150. The four subscales are Severe Combined Abuse (eight items, range of scores = 0–40, suggested cutoff of 1), Physical Abuse (seven items, range of scores = 0–35, cutoff of 1), Emotional Abuse (11 items, range of scores = 0–55, cutoff of 3), and Harassment (four items, range of scores = 0–20, cutoff of 2). The scale has demonstrated convergent and discriminant validity (Hegarty et al., 2005). Cronbach's alpha for the CAS in the current study is .93. Only item 7: “My Partner: Raped me” from the CAS was used to identify interview respondents whose partners had sexually assaulted them. An analysis of the entire sample of 655 women concluded that 41% had been sexually assaulted by intimate partners in response to this CAS item (Tutty & Nixon, 2022).

### Research Procedures: Qualitative Component

Qualitative secondary analysis reuses existing interviews. It has been deemed especially appropriate for dealing with vulnerable populations and sensitive issues (Long-Sutehall et al., 2010, Irwin, 2013). The first and second authors conducted the descriptive secondary analysis of the transcripts following established mainstream qualitative research processes, identifying the major themes, sub-themes, and categories (Graneheim & Lundman, 2004; Miles et al., 2014). First-level coding entails word-by-word scrutiny of the narratives to identify prominent themes and subthemes (Braun & Clarke, 2006). Second-level coding examines the themes and subthemes to identify similarities, differences, and gaps using the constant comparative method (Thorne, 2000). The secondary descriptive qualitative analysis reported here re-analyzed the interviews looking for information regarding IPSV.

## Results

### Forms of IPSV

The categories of IPSV from Bagwell-Gray's taxonomy that the 89 women interviewees did or did not disclose are displayed in Table 2 categorized by their response to the CAS7 question, “how often did your partner rape you?” reduced to yes/no responses. In total, 46 or 51.7% of the women identified having experienced partner sexual violence, the most serious forms being sexual assault (17 or 19%), sexual coercion (12 or 13.4%), and sexual abuse (14 or 15.7%). Forced sexual activity was seldom mentioned as the worst form of sexual violence from partners (3 or 3.4%). More than one-third of those who disclosed IPSV (18 of 46 or 39.1%) described incidents that fit more than one of Bagwell-Gray's categories, most disclosing two types and two women disclosing three. Notably, five women who described being sexually assaulted by partners in the interviews did not indicate having been raped in response to CAS7. Further, only 12 of 38 women (31.5%) who stated that their partners had raped them once or more than once (CAS7), disclosed any sexual assault incidents in the interviews.

**Table 2.** Most Serious Sexual Violence by Extent of Partner Rape (CAS Item 7)

Extent of partner rape (CAS7)	Sexual assault	Sexual coercion	Forced sexual activity	Sexual abuse	None mentioned
None (*n* = 51)	5	7	3	11	25
Once or more (*n* = 38)	12	5	0	3	18
Totals (*n* = 89)	17 (19.1%)	12 (13.5%)	3 (3.4%)	14 (15.7%)	43 (48.3%)

The following sections document the information on IPSV disclosed in the women's interviews, first presenting the least forceful and invasive (intimate partner sexual abuse), then sexual coercion, forced sexual activity, and lastly, the most forceful and invasive form (intimate partner sexual assaults). Because the data in Table 2 refers to the most serious violence mentioned, the numbers in the sections below do not match, as a number of women identified multiple forms of partner sexual violence.

Intimate partner sexual abuse (low force and low invasiveness). As a reminder, this term includes sex outside the primary relationship (i.e., the partner's infidelity), refusing to use condoms, birth control sabotage, and control over sexual decision-making. Twenty-six women (29.2%) described incidents that fit Bagwell-Gray's description of this.

Twenty-five women were aware that their partners were having sex outside of the relationship (one in reference to a lesbian partner). As one woman mentioned, “He was such a womanizer. I never knew it. Must have been one woman after the other. I’d hear rumors. Eventually I found out it was true.” Another commented, “He was physically, mentally abusive, very jealous. He was always messing around with other women. He accused me of having affairs.” A third respondent discovered her partner's affairs by accident:

I came across stacks of pornographic material in his vehicle, tapes, sex toys. His little black book, pictures of women, pictures of him with women. Overwhelming! You can’t believe somebody would do this to you. Somebody you completely trusted.

Seven women described their partners using degrading sexual slurs, mostly calling them “sluts.” Three of these were in the context of accusations of infidelity, such as the following, “Where were you, slut? Were you whoring? Did you go meet somebody?” Another woman who had not described any other examples of IPSV, disclosed the following:

I’d come home and the frickin’ phone's ringing like crazy. “You slut, you whore. Where were you? I knew you were sleeping with somebody.”

Three women described reproductive coercion. One respondent commented, “It was like he wanted me to get pregnant so he can trap me. I did, to please him. Then he never stopped hitting me, never stopped calling me down.” Another two had similar narratives:

He wanted a boy. I was pregnant six months after. I had an abortion because he wanted me to. That was very hard for me; Aboriginal belief is that you give it up for adoption.

It wasn’t long until I’m pregnant again. Always a fight. (So you didn’t want to get pregnant again?) No! But he became very sexually aroused. I’d say, “We can’t do that.” He said, “Don’t tell me I can’t do anything.”

Another two women described their partners refusing to have sex with them, which fits the criterion of not having control over sexual decision-making. As one woman noted, “He was quite cruel. I was rejected in every way. Probably the hardest was being rejected sexually on a continuous basis.” Another commented:

Because I’m chubby, he makes me feel that I don’t need sex. I feel like a hooker with him, because it's to service him. He gags if he has to do anything to me, and if you want to get satisfied, “You do it.” It's like I’m begging for sex with him.

A final woman discovered that her partner had taken pornographic pictures of her while she slept, which he then used to threaten her.

This one night, I had way too much to drink. I remember having someone pull my jacket over my head, saying “We’re going to put you to bed, sweetie”. They brought me into the bedroom, undressed me, and took pictures of me. It was humiliating. The next morning, I remembered the camera and his friend being in the room. I couldn’t figure out the details, so I played like it wasn’t a big deal. We didn’t talk about it. The breaking point was I went out to the bar with some girlfriends. He came up, whispered in my ear, “I have your pictures and I jack off to them every night.” I thought, it's blackmail. He kept talking about what I looked like and what he wanted to do to me.

Although not formally included in Bagwell-Gray's taxonomy of sexual abuse, three women discovered that their partners had sexually abused either their children or their grandchildren. One woman simply commented, “My eldest son found out that my partner had raped his daughter.” The other two recounted the difficult circumstances with respect to discovering that their children had been sexually abused:

“You’re fucking crazy.” He said that about my daughter when she said he had done stuff to her. He tricks everybody. When our daughter got interviewed at the child centre, they were totally concerned. They thought she was credible. Once they met my partner, they threw it out. He was like, “I’m a single dad and when my daughter sees me, sometimes she cries and doesn’t want to spend time with me.” He told them she had made this up because she was mad at him for going to work. Biggest lie ever.

I suspected that he was “bothering” my daughter, but I couldn’t prove it because she was so young. She’d say that he was touching her, and I’d be like “What?” I went to the doctor, but nothing. But I left him when she said that. I never went back.

Two additional women discovered that their partners were making pornographic images of their child or showing pornographic materials to their children.

One day I came home, and this woman said [they] were investigating child abuse. My husband was taking pictures of my daughter in her bathing suit and she complained to some kids at school. I was stunned. The police were hauling out computers. I asked my husband, “Is this true?” He denied it. They said he had threatened to kill my son and me if my [daughter] told. My daughter finally told me they had taken pictures and he would grab the top of her bathing suit, expose her breasts (she was just developing), and try to move the bottom of her panties. He’d never actually touched her, thank God. But she knew it was wrong.

He was using a lot of pornography. I confronted him because of my daughter lying down in front of him, spreading her legs open and saying, “Look at me dad.” I said, get it out of the house. I ended up making him throw them in the garbage. I never realized how much he had. There was two garbage bags full of the pornography.

Intimate partner-forced sexual activity (high force and low invasiveness). Forced sexual activity is defined as “nonpenetrative sexually abusive acts that are physically forced” (Bagwell-Gray et al., 2015, p. 325) such as violence toward a sexual body part that does not entail penetrative sexual acts or being masturbated upon. The authors clarify that “In our taxonomy, for an act of IPSV to meet the criteria of physically forced sexual activity, some degree of physical force is required” (p. 325). Three women (3.4%) described incidents that fit this category.

He would not let me leave. My ribs were recovering from when he’d broken them. He gives me a hug so hard that he re-injures my ribs. Then he gets a knife and says, “Nobody knows where you are. I’m going to kill you and leave you in the ditch after I cut your nipples off and make you swallow them. I’m going to cut your clit off and stab that into your stomach.” I don’t remember how I got myself out. That was the end for me.

Another woman described threats to penetrate her sexually with a hockey stick:

He got abusive because I cheated. I told him, but he was pretty upset. He chased me around the city with his vehicle, called me a slut, and was going to shove a hockey stick up between my legs.

Note that, if the threats in these two examples had been used to force sexual penetration, they would fit the category of sexual assaults.

Another example of forced sexual activity is unexpected physical force during otherwise consensual sex, as described by the following woman who had also disclosed that her partner used sexual coercion:

He was sometimes cruel. One time he just whacked me during sex, slapped me in the face, not very hard, but I was, “What the hell are you doing.” [He said], “It felt right at the moment.” I didn’t like the way he was treating me.

Sexual coercion (low force and high invasiveness). While sexual penetration can occur in the context of sexual coercion, it is not always physically forced. Rather, it can be the result of intimidation, threats to humiliate, withholding essential resources, and verbal coercion. Sixteen women (17.9%) described sexual coercion, the most commonly-noted form being unwanted sexual intercourse (14 women):

Many times, I didn’t want to have sex with my husband, and he made me. No force. He starts touching me. I said, “I don’t want to have sex,” and he keeps touching. [I said,] “I don’t want to have sex,” and he's touching and touching until we end up having sex.

I was maybe 16. After living together a short time, strange things happened, sexually, that I had never been exposed to. I was being coerced into things I wasn’t comfortable with. I didn’t feel I had the right to say no, so I went along and felt degraded and humiliated. I felt like it was my obligation; that he was my partner.

The last three years it started going into sexual abuse. I was abused when I was a child. When I’d tell him I am not interested, he’d be at me, getting mad and I’d have to give in to him just to make him shut up. It made me feel so dirty and I just hated it.

Two women described their partners forcing them to have sex with others, one in the context of sexual exploitation, “I was in the sex trade at the time. When I was being abused, I was told to go out there because he always wanted cigarettes, and he wanted his drugs.”

The other woman commented:

I turned twenty and he took me out. I got absolutely hammered. He invited people back to our place and I went to bed. I woke up to somebody having sex with me. It wasn’t my boyfriend; it was one of his buddies. I remember thinking, “God, what is going on?” I went to get off the bed and he was sitting watching and his other buddy was on the other side of the bed watching. There were other nights like that. He said that it would help our sex life, he would bring other girls, other guys home. He used to tell me our sex life was lacking, and it was okay because he said it was okay.

Intimate partner sexual assault (high force, high intrusiveness). Seventeen women (19%) described being sexually assaulted by their partners, with many being violently raped. One woman disclosed a brutal attack from an ex-partner who broke down her door:

He's banging on the door, saying, “I love you and you shut me out of your life,” and I said, “You need to leave or I’m phoning the police. I have feelings for you, but I wasn’t sure that that's what you wanted.” Suddenly he explodes and says, “LIAR!!” He's screaming, and he kicked the door, and then he's inside and the door's on me, and he's jumping on the door, screaming, “Fucking liar! Liar, liar!” Somehow, I got up from under the door. I remember thinking “If you’re going to hurt me, I’m not going down without a fight.” I grabbed the back of his hair and punched him in the throat. The next thing I know, my feet weren’t touching the floor. He wasn’t a big guy, but I could smell the rage! So he had me up against the wall, his face is purple, and he's looking at me like he wants me dead. The next thing, I was flat on my face on the floor. He was pulled my pants down from behind. I was screaming “no, no, no!” He slammed my head forward and he's *raping* me. I’m on my face and I can hear the police at the front door.

Another woman described the sexual intercourse in the context of other humiliations and abuse:

Physical, emotional, spiritual [abuse], like hell. I would go to school with fat lips and have to cover my neck because I’d been choked the night before with an alarm clock cord, or I’d been peed on. In every single way I’d been degraded, shamed, raped by my partner. I didn’t want to have sex; I didn’t have good feelings. He had done that several times. I understand now that it was spousal rape.

Other women described the brutal details of their partners sexually assaulting them.

He took his pants off, and he ripped my pants off. He got really aggressive and pinned my arms over my head and forced himself on me. He was calling me a whore, and a slut. He thought I was cheating on him. He's spitting in my face—pulling my hair. I started getting really scared. He covered my mouth and nose so I couldn’t breathe, and I started panicking. I started going limp, so he finally took his hand away. As soon as I caught my breath, he did it again. As soon as he took his hand off, I got up. I could barely stand my legs were shaking so bad. Dressed up how he wanted me to dress and went to get on the bed. He told me that I didn’t deserve to be on the bed with him, to get on my knees, and he made me give him oral sex. He grabbed my hair so I couldn’t pull away. He picked me up by my hair and pushed me on the bed. Had sex with me, and then threatened that he wanted to have anal sex. I was really scared. Finally, friends that he called earlier showed up. He told me to get dressed and warned me not to say anything.

I was breastfeeding my baby, and he started touching me. I said, “Get away,” and he persisted to the point where I slapped his hand away. That got him real angry. Next thing I knew he was behind me, choking me with one of his hands around my throat, and his other one on my mouth and nose. He was choking me. I started to panic. I couldn’t fight him because my baby was still feeding, and I didn’t want to disrupt her. I dug my nails into his arm, and he let me go. Then he started raping me. My boys were in the other room, I couldn’t fight, because I didn’t want them to see. My daughter was watching her father. After it was over, it's like nothing happened. For him it was nothing.

He had me stripped totally naked in front of my children, looking for needle marks because he had it in his head that I was doing needles. I never did drugs. I told my children, “Don’t look.” I had to strip while he checked me over. Then he raped me, right there, with my children in the room. It was then I knew I had to get out.

I got pregnant. I was so excited. He smacked me across the face and said, “Whose is it?” I was horrified. I heard the door slam. About half an hour later he came back. He had a lock and he put it on the bedroom [door] so that he could lock it from the outside. He put me in the bedroom and told me I could come out when I told the truth. I kept begging him to believe me. That night he raped me to the point that I was bleeding. I was rushed to the hospital and I lost the baby.

In another extreme example, one woman's partner re-enacted her previous sexual assault:

Through that pregnancy the abuse got worse because he knew it wasn’t his [baby]. He came home one night. My kids were sleeping in the living room, and he started hitting me. When I was eighteen, I got raped by this guy with a gun and he held me for nine hours. I’d told him all about it and he basically did the same thing that guy did to me, except with no gun.

A final woman described another extremely violent incident:

I pulled a butcher knife on him. I wanted to kill him. He went, it was either me or him, so he ended up beating me. He actually had his whole hand up my vagina, and he had a fork at the end of it. Next thing I know, I’m in the hospital. When I finally could open my eyes, guess who was sitting there?

To summarize, almost half of the 89 women described incidents in their qualitative interviews that fit Bagwell-Gray's taxonomy, although the interview schedule did not include questions enquiring about sexual violence.

## Discussion

The women's narratives document significant IPSV across all of Bagley-Gray's categories. Unlike the research of Bagwell-Gray (2021) and Fernet et al. (2021), the interview questions in the current study were not specific to IPSV, and the women chose what or what not to reveal about what we had, perhaps optimistically, deemed their “healing journey from IPV.” That slightly more than half of the women (46 or 51.7%) mentioned some form of IPSV (19% sexual assaults, 17.9% sexual coercion, 3.4% forced sexual activity, and 29.2% partner sexual abuse) without having been explicitly asked is striking, especially since societal taboos about discussing any sexual topics remain (Huff & Rappleyea, 2020). The interviews were conducted about halfway through the longitudinal study, so relationships with the research assistants across the four-year study likely enabled the women to disclose such intimate details.

The IPSV taxonomy categories are similar to the results obtained by Fernet et al. (2021), whose sample was young women in dating relationships with no specified IPV: The current study reported more sexual assaults (19% vs. 14%) and sexual abuse (29.2% vs. 12.7%) but less sexual coercion (17.9% vs. 28.2%) and forced sexual activities (3.4% vs. 7%). Not surprisingly, all categories in the current study were lower than the results from Bagwell-Gray's interviews that used specific IPSV questions for IPV survivors (2021): sexual assaults (19% vs. 50%); sexual abuse (29.2% vs. 96%), sexual coercion (17.9% vs. 68%) and forced sexual activities (3.4% vs. 7%). This clearly identifies the need to use specific IPSV questions when assessing this important variable.

Notably, five women who described sexual assaults from partners did not endorse the CAS item 7 as “ever” being raped. As noted previously, conceptualizing partners’ sexual assaults as “rape” is not necessarily a straightforward process (Howard et al., 2003). Further, only 12 of 38 women (31.5%) of women answered the CAS7 stating that their partners had raped them once or more than once then disclosed partner sexual assaults in the interviews. What if the other 26 women (68%) had been offered the opportunity to describe their partners sexual assaults or other sexual violence more directly?

Further, although the interviews were conducted in 2007–2008, researchers continue to identify that many women do not conceptualize partner sexual assaults as rape (Jaffe et al., 2021; Logan et al., 2015; Tarzia, 2021b; Wright et al., 2021). Tarzia's conceptualization of IPSV using an ecological perspective (2021b) suggests that societal and community values play as much a part as individual beliefs about this. Thus, this misconception must be addressed by multiple initiatives not simply messages to individual women.

Using Bagwell-Gray et al.'s (2015) taxonomy to code IPSV in the women's narratives was relatively simple, especially with respect to the more serious and well-discussed categories of sexual assault and sexual coercion. Her partner forcing her to have sex with other individuals (mentioned by two women) is included as a sexual coercion tactic, but is mentioned by few researchers, an exception being Logan et al. (2007).

The process of coding intimate partner sexual abuse (low force, low intrusiveness) was simple as well. However, seeing this as a form of IPSV may be contentious as a number of these issues are rarely considered as sexual violence, including the partner having affairs, reproductive coercion, using sexual slurs, etc. Although these undoubtedly have psychological consequences on the women, none have criminal consequences according to US legal standards (Bagwell-Gray et al., 2015). The most mentioned example from our interviews was partners being unfaithful, a phenomenon that has rarely been documented as abusive (Pichon et al., 2020; Utley, 2017). The consequences of the male's infidelities certainly impact women's sexuality, whether by eroding trust in the sexual relationships or by introducing sexually transmitted infections from the illicit liaisons (Hess et al., 2012; Utley, 2017). Sexual slurs, especially ones that accuse the women of infidelity in the relationship, were mentioned by three women and degrading sexual comments have been linked to sexual coercion by Starratt et al. (2008) and as occurring in over 90% of the time for both women whose partners forced sex and those who did not (Logan et al., 2007).

The category of forced sexual activity (high force, low invasiveness) was the most difficult to code, necessitating repeated returns to the definition to check whether the incidents were a fit. Even now, we remain uncertain of the fit of our examples. Two involved serious threats toward sexual body parts, however, threats were not specifically mentioned as an example of forced sexual activity in Bagwell-Gray et al. (2015). As this conceptualization was developed as a theoretical description of one quadrant of the circumplex, it perhaps needs to be revisited. Even its description as being “high force, low invasiveness” begs the question of what exactly it might entail.

Issues not currently included in Bagwell Gray's taxonomy or mentioned only in very specific contexts were identified. One woman was sexually exploited (trafficked) by her partner in the context of his sexual violence; labeled as sexual coercion in the current study. While not a new phenomenon, this suggests the need for further research considering this as IPSV (Salina et al., 2016; Thaller & Cimino, 2017). For example, Argento et al. (2014) found high proportions of sex workers in Vancouver had experienced IPSV in the previous six months.

Research examining the impact of using pornography is, similarly, rarely discussed (Brem et al., 2021; Logan et al., 2015; Tarzia & Tyler, 2021) but was included in Bagwell-Gray's taxonomy as an example of sexual abuse. However, using or making pornography with the women's children, mentioned by several women in the current study, is largely absent from the literature on IPSV and would fit with the category of sexual abuse. In addition, sexually abusing one's children or grandchildren could be included as an additional example of sexual abuse.

### Implications for Practitioners

The current study confirms our recommendation from our previous quantitative analysis of IPSV in the total sample of 665 women (Tutty & Nixon, 2022) that those who work with IPV survivors should enquire about sexual assaults and other sexually intrusive behaviors by directly asking about these. Further, given the complexity of issues involved in identifying a partner's behavior as sexual assault, it is essential not to ask a dichotomous yes/no question about whether or not a woman's partner has raped or sexually assaulted her (Logan et al., 2015). However information about IPSV is accessed, it is important to elicit details about the extent and nature of the partner sexual tactics and its effects on the women in counseling sessions. An in-depth discussion of their partner's sexual behaviors that includes Bagwell-Gray's categories of sexual coercion, forced sexual activities, and sexual abuse in addition to sexual assaults is recommended.

Once a partner's sexual violence has been identified, counseling to help women establish positive sexuality in their lives is recommended (Bagwell-Gray, 2019). A small body of literature describes therapy for women whose partners have sexually assaulted them, most of whom have left the relationships (Martin et al., 2007; McFarlane et al., 2005). Studies such as Howard et al. (2003) found that, while women who were raped by intimate partners improved their well-being and coping after community counseling, their functioning was worse at group start and less improved at group finish than women whose partners had physically but not sexually abused them. This suggests the importance of an explicit counseling focus on sexual violence should it be disclosed. Huff and Rappleyea (2020) and Maas-Despain and Todahl (2014) add a unique perspective: Assessing partner sexual violence in intact, committed relationships, with suggestions about possible clinical interventions.

### Implications for Researchers

Researchers are encouraged to examine forms of sexual violence in more detail in their studies of IPV. In our original study (Tutty, Radtke, Ateah et al., 2021; Tutty, Radtke, Thurston et al., 2021), we used the CAS (Hegarty et al., 2005), to capture forms of IPV, not realizing that, although the measure includes three items on sexual violence of a total of thirty and, although all three items are included in the eight that make up the subscale of serious IPV, without a separate subscale on sexual violence, the sexual behaviors of the partners remained obscure. Even a subsequent revision of the CAS (Ford-Gilboe et al., 2016) maintains the current structure of subscales and includes only two items on sexual violence.

Instead, we recommend using sexual violence-specific measures in both quantitative and qualitative studies with a central interest in documenting sexual violence. Even so, most available scales address only sexual assaults and sexual coercion, and, even then, not extensively (Logan et al., 2015). The Revised Sexual Experiences Survey Short Form Victimization (SES-SFV; Koss et al., 2007), as used in Fernet et al. (2021), could be useful. Moreau et al. (2015) added additional items to the SES-SFV to capture issues such as pornography and forced sex with others, which broadens the sexual issues productively.

Similarly, asking explicit questions about partner sexual violence is recommended in qualitative research of women's experiences of IPV. Having interviewers who are highly skilled and knowledgeable about sexual abuse/violence is essential given that their interviewees may not always identify their unwanted sexual experiences as forms of sexual violence or rape, especially in the case of marital rape.

Perhaps some of the anomalies and questions in applying Bagwell-Gray's taxonomy (2021) stem from difficulties applying the constructs of “low” and “high” degrees of force and intrusiveness. In some circumstances, even the “low” use of some behaviors is unacceptable. While these fit nicely into a circumplex, creating a model that is theoretically pleasing, the use of the term “low” implies that these fit society's standards. Bagwell-Gray and colleagues are encouraged to continue their exploration of this useful taxonomy with an eye to clarifying, expanding, and critiquing the model.

### Study Limitations and Strengths

When conducting secondary analyses, one is limited by the nature of the original study, which, in this case, relied on a convenience sample of women from VAW shelters or counseling agencies. Notably, the women were not asked explicitly about any sexual violence directed at them by their intimate partners. As mentioned earlier, that almost half of the women in the study subsequently disclosed some form of partner sexual violence without having been directly asked is notable. Beyond this concern, though, some research assistants in the current study were novice qualitative interviewers and did not follow up when women disclosed sensitive material, likely shutting down more in-depth disclosures.

A strength of the current study is that the women constitute a large sample of IPV survivors from the Canadian prairies with more than half of Indigenous background, a group whose well-being is particularly important in Canada. The women's candor with respect to such taboo topics as partner sexual assaults adds increased urgency to raising awareness of this seldom-discussed aspect of IPV.

## Conclusions

Over 20 years ago, in her introduction to a special *Violence Against Women* issue on marital rape, Yllo (1999) identified the paucity of published research on IPSV. Although this body of research has expanded dramatically since then, especially with respect to partner sexual assaults and sexual coercion, additional research and discussion about forced sexual activity and sexual abuse is needed (Logan et al., 2015).

The current study adds to our understanding of the broad nature of sexual violence and abuse within intimate partner relationships, a topic that has received some attention but that merits considerably more. Despite the fact that the women were not asked explicitly about any sexual violence, the extent to which their narratives provided considerable, disturbing details about the nature and variety of their partner's sexual violence that fit within Bagwell-Gray et al.'s (2015) categories was surprising. One can only speculate about what the women might have disclosed had they been asked directly about IPSV.

Thirty years ago, Diana Russell (1982) wrote that “wife rape cannot and must not be subsumed under the battered woman rubric” (p. 101). We confess that this was the case in our original publications about the Healing Journey project, none of which identified partner sexual assaults separately from IPV (Tutty, Radtke, Ateah et al., 2021; Tutty, Radtke, Thurston et al., 2021). The project was sprawling and complex as conceptualized and, given that the CAS includes several items on sexual violence (but has no separate subscale), we believed that we had addressed this. However, we did not examine these CAS items, only subscale scores. If we had examined the items, the importance of partner sexual assaults would have been clarified immediately. We have since rectified this in a recent publication (Tutty & Nixon, 2022), however, the lack of a mention of IPSV in the early Healing Journey publications suggests that Russell's fears were well-founded with respect to our longitudinal study.

The eclipse of partner sexual violence issues in other research on IPV has likely occurred as well. Additional research is needed with respect to several issues including the extent to which women continue to not perceive IPSA as sexual assaults (Wright et al., 2021) and the nature of IPV and IPSA with respect to women of Indigenous backgrounds (Ogden & Tutty, in preparation), many of whom are more likely to remain with abusive partner (Tutty et al., 2020; McKinley & Liddell, 2022).

Additional research on Bagwell-Gray's taxonomy is needed, in particular, how often these tactics occur, examining whether the existing categories adequately assess the range of sexually abusive behaviors and the mental health consequences of these. In particular, while the category of intimate partner forced sexual activity fits the taxonomy theoretically, it was the most contentious in our coding process, entailing numerous discussions about the fit of the women's examples, especially about what merits a categorization of “high force” and “low intrusiveness.” Further, as noted earlier, expanding the taxonomy to include women's experiences of being sexually exploited by partners and the sexual abuse of women's children (including using/making pornography) should be explored. Nonetheless, Bagwell-Gray's taxonomy is an important addition to discourse about the broad and serious nature and consequences of sexual violence in intimate relationships.
